# Cancer in Africa
*The Long Fox Memorial Lecture, University of Bristol, 1st November 1962.


**Published:** 1963-01

**Authors:** J. N. P. Davies


### CANCER IN AFRICA*

BY

J. N. P. DAVIES, M.D. (BRISTOL)

For his friends to combine to found this lecture and to confine it so strictly within the orbit of this city shows that Dr. Long Fox was proud to be a citizen of no mean city and of its Medical School which he had adorned. So to an exile, also proud of his native city and of the University which nurtured him, I thought it appropriate that I should honour Dr. Fox as a teacher in this Medical School by trying to honour my own teachers, as Hippocrates said we should do. Happily many are still with us and to them, and to all my other teachers I would like to express my gratitude, for even those with whom I came little in contact have helped me in various ways, if unknown to them. One winter's day before my finals I went on my only ward round with Dr. Orr-Ewing. He was so moved, I know not by what, to speak of typhus fever and the hideous hallucinations which accompany it. He sent me to that famous book of a physician with Bristol connections, Murchison's *Continued Fevers of Great Britain* which I read with fascination. Years later I was standing by the bedside of an almost comatose lady missionary. Africa being what it is, I had already treated her as a case of cerebral malaria and was now, I know Professor Perry will forgive me, taking a history from her companion and going on to examine her. I heard of the fever and rigors, of the severe headache, and then her companion said "and Doctor she said saw hideous shapes at the foot of the bed and tried to throw her pillow at them". The cobalt sky of Africa immediately became the pearly sky of a Bristol winter day and I was back listening to Dr. Orr-Ewing. I thought "this must be typhus" and so it proved. She thanked me on her recovery but she also thanked, through me, my teachers.

But it is of three of these who have passed to their reward that I wish particularly to speak. The first, that giant amongst men, Professor T. B. Davie, who gave me a life-long interest in pathology for which I can never be too grateful. Then there is the late Dr. H. H. Carleton, for I was one of the first Infirmary students to brave the bracing river smell of the Bristol General Hospital and clerk for the famous firm of "Carleton and Perry". Professor Perry taught us, as he still does, the rigours of scientific medicine and how many are the generations of us who are grateful to him, while Dr. Carleton in his gentle precise way taught us the same, but also something of the spirit and philosophy of medicine. From him I learnt that it was the constant duty of the physician to observe and consider, using that finest of all research tools the human brain and that this was always available for service despite the absence of the apparatus, instruments, and gadgets indispensable in modern medical research. From the firm of Carleton and Perry I passed to clerk for that strange, difficult, often wilful, person, the late Dr. A. T. Todd. From him I learnt much, for though he would insist that I read and learnt orthodox medicine, he would make me read disturbingly critical papers and not only in English. From him I learnt a deep distrust of so much that passes for fact in the medical literature as well as a very deep interest in the problems of cancer and its treatment.

With these influences upon me it was perhaps natural that when, after various vicissitudes, I should find myself in Uganda, I was immediately interested in cancer in that milieu amongst the native African people. Even if apparatus and instruments were few and old, there was much, as Dr. Carleton had urged, to note and speculate upon. For there was something very odd either about the patients themselves or my

* The Long Fox Memorial Lecture, University of Bristol, 1st November 1962.

clinical colleagues. It is perhaps natural for a pathologist to suspect oddity in his clinical colleagues rather than in the patients, but why in the laboratory and autopsy room did we see such a curious collection of cancers quite unlike those familiar to me in Bristol? Lung, stomach, colonic and rectal cancers were rarely seen while there was a steady stream of primary liver cancers, of the curious skin tumour known as Kaposi's sarcoma, and many other tumours which I had rarely seen or heard of in my home town. For a number of years we studied small groups of cases but it became apparent that if we were to gain real knowledge we must make a radically different approach to anything which had been done in the past. Official statistics were useless, and biopsy and autopsy figures too selected, to give us the total cancer picture.

But there was a particular set of circumstances in Kampala which made this new approach possible. Though there were five hospitals in the town, governmental or missionary controlled, the relations between the staff of these various hospitals was close and cordial, a relationship much fostered by the Uganda branch of the British Medical Association. Furthermore all these hospitals depended on the same ancillary diagnostic units in radiology and pathology. Moreover in the absence of private hospitals all general practitioners depended for their consultations on these same services and all the specialised medical facilities of Uganda were centred in Kampala.

It therefore seemed to us that if we could register all cases of cancer attending the various hospitals of Kampala and in the ancillary diagnostic units we would be able to obtain details of all cancer patients who sought medical attention. All the clinicians in the town generously cooperated and allowed us access to their cases, even though one warned us we were wasting our time as there was hardly any cancer in Kampala to register. We first had to stop one loophole. This was that patients coming with very advanced cancer might not be admitted to hospital because of the severe bed pressure and because in the absence of radiotherapy we could offer no treatment. So for several months in 1951 we kept a close check on the out-patients' department. Rather to our surprise we found very few such patients because our clinical colleagues made a practice of admitting suspected cancer cases forthwith for a more thorough examination in the wards than was practicable in a crowded out-patients' department. None the less there were a few such patients and clinicians agreed to notify us of their details. By the end of 1952, we had registered 383 cases of cancer, 348 in Africans, so after all there was plenty of cancer in Kampala.

For two years we continued to use a retrospective survey system, but when we assessed the results it was clear that too often vital information was missing, such as age, tribe, address, or even sex. With aid from the British Empire Cancer Campaign we were able to provide a cancer registrar and put the survey on a current basis. We also discovered two important things, the first was that we could ascertain the ages of our patients with reasonable accuracy, especially if the patient could be made to think it important, by the use of dated local events. If I told you that one of my first memories was of ambulance trains passing full of khaki clad soldiers, that in my second or third year at school I could remember armed men passing on lorries and was told it was a general strike and that I qualified in medicine just before the last war started, you could, if you wished, estimate my age fairly closely. This was the method we used and it proved accurate certainly to a decade and usually to a quinquennium. Secondly we found that, quite contrary to expectations, it was easy to follow up the majority of our patients. In a largely preliterate society there is little to do in spare time but talk, and by concentrating on a patient's friends, relatives and social background, it proved quite easy to follow patients. Someone always knew what had happened to them. Paradoxically it is probably more difficult to disappear in central Africa than in a Bristol suburb. So we appointed a trained African social worker to see and follow up patients. The only demand on our busy clinicians was for out-patients' cards and access. We nosed out all the rest. Our best help came not from the senior clinicians, conscious of differential diagnoses and reluctant to commit themselves till certain, but from sisters, nurses and students. These were willing to say "register the case in bed 10. He's got a lump. Dr. So and So doesn't know what it is but I think it might be cancer". By the time Dr. So and So had made up his mind we had the case registered, and of course we wanted to register on suspicion for we could strike out any non-cancerous case later. Where histological confirmation was not available the case notes and data were individually considered by the Cancer Research Committee before being accepted.

In all this we had the constant help and advice, and criticism, of Professor John Knowelden of the University of Sheffield, and on these lines we continued till December, 1960. Various cross checks convinced us that we were in fact registering virtually all patients with cancer who sought relief in Kampala. We knew their addresses but the difficult problem was to get satisfactory demographic data. At the previous census in 1948, the population was only enumerated in the following age groups: 0–1; 1–4; 5–15; 16–45 and over 45, corresponding to the infants, toddlers, school children, working population and the old, for only 11 per cent of the population were over 45 years. These groupings were quite useless for our purposes. But after we had conducted three pilot surveys to prove that it was possible the Uganda Government agreed in its 1959 census to try to obtain actual ages. This was done in the environs of Kampala in the county of Kyadondo with a population of over 200,000 in 1959, by means of a 25 per cent sample census, this sample size having been predicated by means of the pilot surveys. The results were satisfactory.

We thus had the information we required to work out the cancer incidence rates for a tropical population for the first time. We had all the cancer patients who sought medical attention from Kyadondo county and we had the population and the age groups and Dr. Knowelden visited us to work out the results.

Before discussing these let me say a little about the population of Kyadondo. Most are rural dwelling peasants with a number who work in Kampala, supplemented by those, usually immigrants from other areas, who live in "shanty towns" on African freehold land in the peri-urban areas. The population structure differs markedly from that of Britain in that it is a youthful population with far fewer people over the age of 45 years. The population pyramid thus differs markedly from ours. A bulge in the twenties is due to immigration, but we were puzzled, as we were in the pilot surveys, by a population drop in the early teens. This was explained by my colleague Professor R. F. A. Dean who found that Kyadondo is a food deficit area where a family cannot grow all the food it needs. So if you have rapidly growing children there, you send them away to friends or relations in areas where food is more easily obtained. But the different population structure posed problems when it came to comparing our findings with those of other countries. Fortunately a meeting of the African Cancer Committee of the U.I.C.C. in Kampala proposed the use of an "African Standard Population" based on a number of demographic surveys in trans-Saharan Africa. Dr. Knowelden then calculated our data, and those from the U.S.A., Norway and South Africa, on the basis of this standard population.

And immediately we got a shock. Our previous experience (published in 1958) and the results in other countries had led us to expect that the cancer incidence in Africans would rise more and more steeply with age as it does in Europe and the U.S.A. Rather to our dismay it did not. We could find no hint of a rise with age above the ages of 55–64 in males and 45–54 in females.

We were reluctant to accept this, so we went back to overhaul our survey mechanism but we could find nothing to suggest that we had tended to under-register elderly cases, in fact just the opposite, the older people got the more they tended to over-estimate their age. We then assumed that older people just did not attend hospital when they had cancer. But the more we probed into it the less likely did this appear to be the explanation. In fact the "over 45's" attended hospital in considerable excess of the proportion they bore to the total population and were autopsied to a greater extent. Moreover in the autopsies of older persons we rarely found unsuspected cancers. Treatment is free in Kyadondo, the county is small, the people appreciate and readily seek attention, many for trivial complaints, and communications are good. Moreover the disparity is so great that we must have been, in some age groups, missing three or four or more times the number of cases we were registering each year. In a well doctored area this seemed unlikely. Moreover there were some special local conditions which should have brought in patients. A particularly important cancer in Kampala is the curious tumour known as Kaposi's sarcoma. My colleague Mr. John Cook discovered that these could be successfully treated by intra-arterial injections whereas previously all that we could offer was amputation. This caused great local interest and patients with this disease flocked in from far and near, but no more from Kyadondo than we had been getting. For these and other reasons we believe that the true state of affairs is as I have depicted in Kyadondo, and we are encouraged in this belief by a consideration of the reports of a rather similar survey in the African population of South Africa for they are essentially the same as our findings only ours show a more exaggerated state of affairs. They found the cancer incidence rates in Africans in Johannesburg did not rise above the age of 65, there was no evidence that this was due to failure to attend hospital and for reasons which I shall come to, they thought it might be due to an actual freedom from cancer in the older age groups.

This is why I have chosen to talk on this subject in Bristol, not just as a tribute to my teachers whose inspiration you can detect in this, but because I think it poses problems of direct relevance to you. We are so used to the evidence that the incidence of cancer rises steeply with age and with the corollary that follows from this, namely that if we all lived long enough, all of us would develop cancer, that many have accepted that cancer is a biologic effect of age. This is supported by a great weight of evidence but in all this evidence there is one flaw. It is all derived from advanced industrialised urbanised communities. Even in these, however, we know the rural rates lag behind the urban rates and we also know that this is not due to diagnostic failures. Now two African surveys have failed to show this rise; this failure is most marked in a rural peasant population not exposed to urbanisation and industrialisation, and it is present if less marked in an African population only partly exposed to these influences. Perhaps then the bulk of cancer in industrialised communities is the result of cumulative exposure to carcinogenic influences? We accept this for some cancers, perhaps it is true of most, and thus much of cancer in older people is theoretically preventable. Surely this is a goal worth striving for by further investigations into the cancer situation in non-industrialised communities.

Even if our total conclusions in this matter are wrong (and we know well the deficiencies in our own material and the gaps in our knowledge) it still remains that we have demonstrated beyond question that in Africa there is a great difference from Europe and America in the sites in which cancer occurs in the body and the types which occur. Thus in the African material there is a great lack of cancer of the whole gastro-intestinal tract below the oesophagus, of breast cancers, of lung cancers, of tumours of the central nervous system, and of many other types. Instead we have a gross excess of such tumours as primary liver cancers, carcinoma of the penis, tumours of the jaws and external eye tissues, as well as large numbers of tumours such as Kaposi's sarcoma which are extreme rarities in Britain. I would say you would see more examples of this tumour in one year in Kampala than you could expect to see in Bristol in a century. We also have some tumours such as the now notorious lymphomatous tumour of African children.

Perhaps you will bear with me if I say a little more about this cancer, the focus of so much current interest. For it is a story in which you can take a particular pride in that we owe our recognition of it, and of its curious features and its possible unique significance, entirely to men educated in the West Country. We now know that this tumour has been occurring for at least the last 60 years in Uganda but other than an odd comment about its distinctive features, it had not been recognised. Our cancer survey had been running for several years when we received a letter from Professor Waldron Smithers of London inviting us to show an exhibit of our work at the forthcoming International Cancer Congress in London (1958). A meeting of the Cancer Research Committee was held and we agreed we could not refuse this generous offer. So we decided to show a panel on the general cancer situation, ulcer cancer of the legs, Kaposi's sarcoma and jaw tumours; in the latter we proposed to show some of our horrific adamantinomatous tumours. My brother, Dr. A. G. M. Davies, a Bristol graduate, then Specialist Radiologist to the Uganda Government, went to his department to look out suitable X-rays. Some while later he telephoned to say that he had looked through his jaw X-rays and had discovered eleven cases which, when he saw them all together, convinced him that he was seeing what could only be a multicentric sarcoma arising in the marrow of the jaw bones in children. I said that I had never heard of such a thing but he gave me the case numbers and I said I would review the slides. I asked the cancer registrar to look them out. As I was waiting for them the door was flung open and in came Mr. D. P. Burkitt, Specialist Surgeon to the Uganda Government, and said that he had two cases in children in the wards which he thought were a previously unrecognised multicentric sarcoma of the jaw bones and he now recollected having seen others. I said that if he went to the X-ray department he could see the X-rays of eleven other cases. And so was made a discovery which may have important consequences and so began a period of collaboration and intense work which still goes on and in which I have been privileged to take some part.

At first we were rather bemused by the jaw tumours and wondered if they were due to radioactivity, but Mr. Burkitt soon showed that all cases with jaw lesions had tumours in other organs. It was found that these were of the same type, and that many children had such tumours without jaw lesions. So we re-examined all cases of cancer in children in our biopsy and autopsy material and I went through a humiliating period as diagnosis after diagnosis that I had confidently made in the past was ruthlessly swept aside. The clarified picture was an extraordinary one. Firstly we found that this was a very uniform lymphomatous tumour with a highly characteristic appearance, responsible for just one half of all malignant tumours seen in African children in Uganda, though it also affected children of other races. Clinically and pathologically it was worked out that only 50 per cent of cases had jaw lesions. It was then shown that this was the commonest tumour in African children affecting the following sites—jaw bones, salivary glands, thyroid, heart, liver, kidneys, adrenals, ovaries and testes, and that bilateral organs were often bilaterally affected. On the other hand, the lungs rarely showed tumours and the peripheral lymph nodes were only slightly affected, the brain and skull bones very rarely. This curious distribution suggested that we were dealing with a multicentrically originating cancer rather than a metastasing one and Mr. Burkitt worked out a whole series of clinical patterns of presentation.

Then again it was clear that it did not affect very young children but the main peak was about the age of 5–9 years with a few cases in adults over the age of 15. But right from the start a very curious thing had been noted. So many of the cases came from the sparsely inhabited northern plains of Uganda but none from the densely populated south west of Uganda, from whence came so many children with other tumours. At first sight this seemed to indicate a very curious geographic distribution. But we were very cautious at first in accepting this because Mr. Burkitt had been a surgeon in north Uganda where his prestige and influence were immense.

Perhaps these unfortunate children, none of whom returned, were being sent down from the north because of Mr. Burkitt's prestige, but no one was bothering to send them in from other areas. But on the spot enquiry confirmed this curious distribution. Mr. Burkitt has now mapped the distribution over a vast expanse of trans-Saharan Africa and a remarkable pattern has now emerged, that this is a climatically conditioned tumour which does not occur in the colder upland countries. In the equatorial belt the limiting altitude is about 5,000 feet, in the Rhodesias 3,000 feet and in the coastal plain on East South Africa it is 12–1500 feet. We know of no other cancer so curiously limited and it appears not impossible that this tumour might be induced by a virus or similar agent and moreover this might be spread by an insect vector. These possibilities are being investigated. This curious geographic distribution seems a unique manifestation. The disease is not a leukaemia though tumour cells may be found in the marrow of many bones but we wondered if this might be a biological variant of leukaemia.

The implications of this are immense and far beyond the scope of one lecture, for not even now have I exhausted the catalogue of fascinating possibilities raised by investigations in Africa. In Bristol you have a famous registry of bladder and bone tumours. The types of tumour which occur in the bladder in Uganda are quite different to those which occur in Bristol in their respective proportions. Why should this be? The possibilities of research in these fields seem endless. But what I wish to emphasise is that these are not just interesting traveller's tales from a far country, but problems of direct and immediate interest to you. You and I are, or will be, the *corpora vile* of the Registrar General one day. In Africa there is evidence of a very different cancer pattern from that exhibited in Britain, and in particular, as the Johannesburg workers recognised, a distinct absence of just those cancers which are particularly responsible for those rapidly rising incidence rates with age which are such a well known feature of cancer in this country. Can these cancers be prevented? That is the great question I would like to leave with you tonight. Uganda, like other African countries, is a poor country with immense problems and scant resources. She can ill afford the time and effort to study these problems. But surely they are of direct concern to you and a legitimate extension of the cancer research interests of this country?

And so I must conclude this small tribute to my city and university and medical school and to the memory of Dr. Long Fox and to my teachers. Were they with us I hope Professor Davie would feel some pleasure that the interest in pathology he strove to promote in his student had not been lost, that Dr. Todd would feel similarly in that the flickers of interest in cancer which he tried to fan still burnt with a steady flame, and the Dr. Carleton would have felt that at least his student had tried to live up to his teaching, that he had tried to observe and use his critical faculties in an environment without the elaborate gadgets of modern medical research. For they all share in that continuous undying stream of teacher to the taught that is the great pride of every real teacher and as Kipling wrote when Dr. Long Fox could read it:

> "for their work continueth
> broad and deep continueth
> greater than their knowing."

